# Urinary PGE-M in Men with Prostate Cancer

**DOI:** 10.3390/cancers13164073

**Published:** 2021-08-13

**Authors:** Maeve Kiely, Ginger L. Milne, Tsion Z. Minas, Tiffany H. Dorsey, Wei Tang, Cheryl J. Smith, Francine Baker, Christopher A. Loffredo, Clayton Yates, Michael B. Cook, Stefan Ambs

**Affiliations:** 1 Laboratory of Human Carcinogenesis, Center for Cancer Research, National Cancer Institute, National Institutes of Health, Bethesda, MD 20892, USA; maeve.bailey-whyte@nih.gov (M.K.); tsion.minas@nih.gov (T.Z.M.); howet@mail.nih.gov (T.H.D.); tangw3@mail.nih.gov (W.T.); cheryl.smith2@nih.gov (C.J.S.); francine.baker@nih.gov (F.B.); 2 Division of Clinical Pharmacology, Department of Medicine, Vanderbilt University Medical Center, Nashville, TN 37232, USA; ginger.milne@vumc.org; 3 Cancer Prevention and Control Program, Lombardi Comprehensive Cancer Center, Georgetown University Medical Center, Washington, DC 20007, USA; cal9@georgetown.edu; 4 Center for Cancer Research, Department of Biology, Tuskegee University, Tuskegee, AL 36088, USA; cyates@tuskegee.edu; 5 Division of Cancer Epidemiology and Genetics, National Cancer Institute, National Institutes of Health, Bethesda, MD 20850, USA; cookmich@mail.nih.gov

**Keywords:** prostate cancer, aspirin, health disparity, prostaglandin E metabolite, cyclooxygenase, inflammation

## Abstract

**Simple Summary:**

Elevated levels of urinary prostaglandin E metabolite (PGE-M), a marker of inflammation, have previously been associated with cancer incidence and metastasis. Studies investigating PGE-M in prostate cancer are lacking even though chronic inflammation is a candidate risk factor for the disease. We investigated the association of PGE-M with lethal prostate cancer. We measured PGE-M in the urine of men with prostate cancer and in men without prostate cancer (population controls). Our participants included African American and European American men. Because African American men die more frequently from prostate cancer than European American men, we investigated whether high PGE-M may contribute to the increased mortality among African American prostate cancer patients. We did not observe a relationship between PGE-M and prostate cancer aggressiveness or prostate cancer-specific mortality in our study population, neither in the combined cohort nor in the race/ethnicity stratified analysis. Interestingly, however, we observed a significant relationship between high PGE-M and all-cause mortality in African American men with prostate cancer. Yet, there was no association between high PGE-M and all-cause mortality when these men were regular aspirin users.

**Abstract:**

Urinary PGE-M is a stable metabolite of prostaglandin E2 (PGE2). PGE2 is a product of the inflammatory COX signaling pathway and has been associated with cancer incidence and metastasis. Its synthesis can be inhibited by aspirin. We investigated the association of PGE-M with lethal prostate cancer in a case–control study of African American (AA) and European American men. We measured urinary PGE-M using mass-spectrometry. Samples were obtained from 977 cases and 1022 controls at the time of recruitment. We applied multivariable logistic and Cox regression modeling to examine associations of PGE-M with prostate cancer and participant survival. Median survival follow-up was 8.4 years, with 246 deaths among cases. Self-reported aspirin use over the past 5 years was assessed with a questionnaire. Race/ethnicity was self-reported. Urinary PGE-M levels did not differ between men with prostate cancer and population-based controls. We observed no association between PGE-M and aggressive disease nor prostate-cancer-specific survival. However, we observed a statistically significant association between higher (>median) PGE-M and all-cause mortality in AA cases who did not regularly use aspirin (HR = 2.04, 95% CI 1.23–3.37). Among cases who reported using aspirin, there was no association. Our study does not support a meaningful association between urinary PGE-M and prostate cancer. Moreover, PGE-M levels were not associated with aggressive prostate cancer. However, the observed association between elevated PGE-M and all-cause mortality in AA non-aspirin users reinforces the potential benefit of aspirin to reduce mortality among AA men with prostate cancer.

## 1. Introduction

Chronic inflammation has been implicated in prostate cancer etiology and progression [1,2,3]. The pro-inflammatory cyclooxygenase (COX) pathway, where arachidonic acid is converted to bioactive prostaglandins and eicosanoids via the COX-1 and COX-2 enzymes, is linked to elevated systemic inflammation [4]. Upregulated expression of the COX-2 enzyme in cancer cells has been associated with metastatic prostate cancer [5,6,7], but the clinical significance of COX-2 inhibition for the treatment of prostate cancer remains uncertain [8].

Prostaglandin E2 (PGE2) is the most abundant prostaglandin synthesized by the COX pathway. In colon cancer, this prostaglandin is the key mediator of the oncogenic effects of COX-2 [9]. PGE2 promotes cancer cell survival and metastasis through stem cell expansion and inhibition of the p53 pathway in colorectal cancer [10,11]. Endogenous PGE2 production can be estimated by measuring its major metabolite, urinary PGE-M (11α-hydroxy-9,15-dioxo-2,3,4,5-tetranor-prostane-1,20-dioic acid) [12,13]. Elevated PGE-M has been associated with a risk of cancer in many sites, including colorectal cancer [14], postmenopausal breast cancer [15], pancreatic cancer [16], and gastric cancer [17]. This pattern is not universally described, however, and a null association has been reported for ovarian cancer [18]. Additionally, the use of the COX-2-specific inhibitor, celecoxib, did not improve survival in prostate cancer, as reported in the STAMPEDE trial [8]. Elevated PGE-M has been associated with metastasis of breast cancer to the lung [4] and colorectal metastasis in mouse models [10,14]. To date, there has been a lack of studies investigating the relationship between PGE-M and prostate cancer despite strong rationale, with chronic inflammation being linked to the disease as a candidate risk factor [2,19]. 

The anti-inflammatory drug aspirin is now recommended by the US Preventative Services Task Force for the prevention of colorectal cancer [20,21]. Aspirin can inhibit PGE-M levels, and the ASPIRED trial reports utility for measuring urinary PGE-M as a biomarker of aspirin effectiveness in the prevention of disease recurrence [22]. Aspirin has also shown chemopreventive effects against aggressive prostate cancer in high-risk populations [23,24] and lethal prostate cancer in general [25,26]. 

With strong prior evidence suggesting inflammation as a risk factor of aggressive prostate cancer, this study aimed to identify if elevated urinary PGE-M levels are associated with adverse survival outcomes in men with prostate cancer. Furthermore, we aimed to identify if aspirin use may influence these survival outcomes.

## 2. Materials and Methods

### 2.1. Study Population

The NCI–Maryland prostate cancer case–control study has been previously described [24,27]. The study was initiated to test the primary hypothesis that environmental exposures and ancestry-related factors contribute to the excessive prostate cancer burden among African American (AA) men. Prior to interview, all subjects signed informed consent for participation. All study forms and procedures were approved by the NCI (protocol #05-C-N021) and the University of Maryland (protocol #0298229) Institutional Review Boards. Research followed the ethical guidelines set by the Declaration of Helsinki. Cases were recruited at the Baltimore Veterans Affairs Medical Center and the University of Maryland Medical Center through arrangements with physicians. Controls were identified through the Maryland Department of Motor Vehicle Administration database and were frequency-matched to cases on age and race. This article follows the STROBE guidelines for the reporting of observational studies. See Supplementary Methods for exclusion/inclusion criteria, questionnaire, and biospecimen information.

### 2.2. Laboratory Assay for Urinary PGE-M Measurement

Urinary 11a-hydroxy-9,15-dioxo-2,3,4,5-tetranor-prostane-1,20-dioic acid (PGE-M) was measured by the Eicosanoid Core Laboratory at Vanderbilt University Medical Center (Nashville, TN, USA). See Supplementary Methods for more details about this assay and its quality control performance. Measurement of urinary PGE-M has been established as a reliable reflection of circulating prostaglandin E2 [28].

### 2.3. Assessment of Aspirin Use

Our survey evaluated aspirin use with the following question: “Have you taken aspirin or aspirin-containing compounds (such as Bufferin, Anacin, Ascriptin, Excederin) regularly—at least one pill per week for 2 months during the past 5 years”, with responses no, yes, or do not know.

### 2.4. Statistical Analysis

Data analysis was performed using the Stata/SE 16.0 statistical software package (StataCorp). All statistical tests were two-sided. An association was considered statistically significant with *p* < 0.05. For analysis, we assessed PGE-M as either a continuous measure or assigned PGE-M values to quartiles (Q1–Q4, Q1 being the lowest, Q4 being the highest) and median (≤median/>median) with cutoff points determined using the distribution of PGE-M values among all controls. PGE-M data analyzed as a continuous measure were log2 transformed. The non-parametric Mann–Whitney test was used to determine differences in PGE-M levels across cases and controls. Furthermore, cases were assigned to risk groups according to National Comprehensive Cancer Network (NCCN) Risk Score classification, which stratifies patients into pretreatment recurrence risk groups according to the clinical tumor stage, biopsy Gleason score, and serum prostate-specific antigen level [29]. We condensed these risk groups into 4 categories (low, intermediate, high/very high, and regional/metastatic).

Unconditional logistic regression models were used to calculate adjusted odds ratios (OR) and 95% confidence intervals (CI) to assess the association of PGE-M with either use of aspirin in cases and controls, case status, or the NCCN risk score in cases. We adjusted for the following potential confounding factors: age at study entry, body mass index (BMI), diabetes, aspirin use, education, family history of prostate cancer, self-reported race, smoking history, treatment, disease stage, and Gleason score (see Supplementary Methods for more information). To test for heterogeneity of odds ratios, we applied the Breslow–Day test. To test for statistical interactions, we applied the multivariable logistic regression model with and without the interaction term and examined significance with the likelihood ratio test. A *p* < 0.05 was considered as statistical evidence for effect modification.

We applied the Cox regression model to estimate adjusted hazard ratios (HR) and 95% confidence intervals (CI) for all-cause mortality and prostate cancer-specific mortality. Median survival follow-up for cases was 8.4 years. In the analysis of all-cause mortality, median follow-up time to death from any cause was 4.52 years for AA men and 5.99 years for European American (EA) men. In the analysis of prostate cancer-specific survival, median follow-up time to death from prostate cancer was 2.75 years for AA men and 7.7 years for EA men. We adjusted for potential confounding factors (defined in Supplementary Methods). We calculated survival for cases from date of diagnosis to either date of death or to the censor date of 31 December 2018. We confirmed non-violation of the proportionality assumption based on the goodness-of-fit test using Schoenfeld residuals. For survival analysis with the Kaplan–Meier method, the log-rank test was used to examine differences in all-cause and prostate cancer-specific mortality according to PGE-M levels.

## 3. Results

### 3.1. Clinical and Demographic Characteristics of Participants in the NCI–Maryland (NCI–MD) Prostate Cancer Case–Control Study 

Demographic characteristics of the enrolled subjects are shown in Table S1, together with the disease characteristics of the cases. The study enrolled 977 cases (490 AA and 487 EA) and 1022 population controls (479 AA and 543 EA) from the greater Baltimore area in Maryland. Race/ethnicity was self-reported as part of the eligibility screener and within the questionnaire.

### 3.2. Urinary PGE-M Levels Do Not Differ between Men with Prostate Cancer and Population-Based Controls

**Figure 1:**
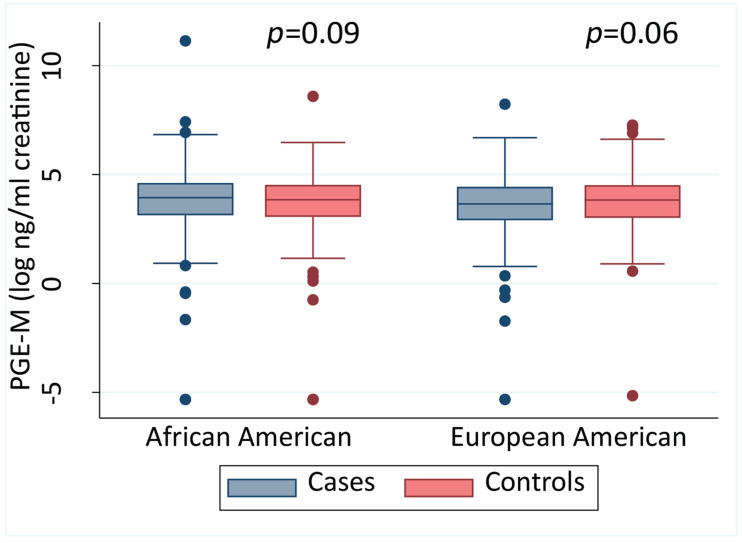
Urinary PGE-M levels in African American and European American men. Urinary PGE-M levels in cases and controls stratified into African American and European American men shown as a continuous measure, stratified by race. A two-sided Mann–Whitney test was applied for statistical significance testing. The error bars represent the 95% CI.

We measured urinary PGE-M in samples that were obtained from 975 cases and 1020 controls at time of recruitment. We then investigated whether urinary PGE-M differed between men with and without prostate cancer. Stratified by self-reported race, we did not observe consistent differences between cases and controls for the AA and EA men (Figure 1).

### 3.3. High PGE-M Is Associated with Moderately Decreased Odds of Prostate Cancer in EA Men in the Multivariable Analysis

We used unconditional logistic regression to further analyze the relationship of PGE-M with a prostate cancer diagnosis and estimated the odds of having prostate cancer when PGE-M is elevated. The adjusted OR for prostate cancer was 0.86 (95% CI 0.72–1.04) for men with high (>median) PGE-Mcompared to low PGE-M (≤median) (Table 1), i.e., no statistically significant association of PGE-M levels with case status in the combined cohort. Because the COX signaling pathway may affect AA and EA prostate cancer patients differently, we stratified the analysis by race/ethnicity. Here, a moderate but statistically significant inverse association between high PGE-M and having prostate cancer was observed among EA men (OR = 0.76, 95% CI 0.59–0.99) but not AA men (OR = 1.00, 95% CI 0.75–1.31). Heterogeneity of the odds ratios was further demonstrated using the Breslow–Day test (*p* = 0.03). Our finding for EA men was confirmed when PGE-M was treated as a continuous variable (OR = 0.88, 95% CI 0.79–0.98). This observation would suggest a moderate protective rather than a deleterious effect of high PGE-M in association with prostate cancer development in EA men.

Elevated PGE-M has previously been associated with lung and colon cancer metastasis [4,14], so we investigated the association between PGE-M, aggressive disease, and metastasis. We assigned men with prostate cancer into NCCN risk groups as described under Methods and shown in Table S2. There was no association between high PGE-M (>median vs. ≤median) and the risk score classification for localized disease and metastatic disease, indicating that urinary PGE-M levels do not define intrinsic prostate cancer aggressiveness (Table 2).

### 3.4. PGE-M and Prostate Cancer Mortality

We next examined if there is an association between PGE-M and survival outcomes in our case population. As of the end of 2018, there have been 246 deaths in our case population, of whom 27% had a prostate cancer as the recorded primary cause of death (n = 66).

Applying a multivariable-adjusted Cox regression model (Table 3), we report no significant association between PGE-M levels and all-cause mortality among men with prostate cancer in the unstratified analysis when PGE-M levels were coded as both a dichotomized (≤median compared to >median) and continuous measure. The observation remained consistent even when our analysis was further stratified by self-reported race. Prostate cancer-specific survival is a key outcome measure for prostate cancer patients. Using a multivariable Cox regression model, we found that high PGE-M was not associated with lethal prostate cancer, although the number of prostate cancer deaths was limited (Table 4) (Figure S1 for unadjusted Kaplan–Meier graphs). Similar relationships were observed when we used continuous PGE-M data in the survival analysis (Table 4).

### 3.5. Aspirin Use Attenuates the Association between Elevated PGE-M and All-Cause Mortality in Prostate Cancer Patients

Aspirin has been shown to inhibit PGE-M biosynthesis [22], so we explored the possibility of an interaction between aspirin use and PGE-M on survival. No such interaction was found in the analysis of all-cause survival (*p* = 0.11) (Table 5) but stratification of cases by aspirin use status (yes/no) revealed disparate outcomes. There was a statistically significant association between high PGE-M and all-cause survival in the cases who reported no aspirin use. For the cases who reported using aspirin, there was no significant relationship (HR = 0.92 95% CI 0.63–1.34). However, when stratified by self-reported race, the association between high PGE-M and all-cause mortality remained significant only in AA men who did not use aspirin (HR = 2.04, 95% CI 1.23–3.37) (Table 5). This finding is mirrored in the analysis of PGE-M as a continuous variable with a positive association with all-cause mortality for non-aspirin users (HR = 1.27 95% CI 1.03–1.57) and no association with all-cause mortality of aspirin users (HR = 0.85 95% 0.70–1.02). 

## 4. Discussion

In this retrospective case–control study, we report no difference in urinary PGE-M levels between cases and controls. We observed no association between elevated PGE-M and metastatic disease nor prostate-cancer-specific mortality. Of note, however, we report a significant association between elevated PGE-M and increased all-cause mortality in AA men with prostate cancer when they did not take aspirin. This association was not observed in AA men who had reported aspirin use, suggesting that aspirin may reduce all-cause mortality for AA men with prostate cancer. This is potentially of clinical importance as these results support a role for aspirin as a chemopreventive agent against mortality in men with prostate cancer. However, the hypothesis that the chemopreventive mechanism of action of aspirin is via PGE-M in prostate cancer is not supported by this study, as we did not find that aspirin use inhibited PGE-M formation in AA men with prostate cancer (Table S3).

Dysregulated COX signaling and PGE2 production have been observed across many tumor types [6,30,31,32,33]. Elevated PGE-M, reflecting in vivo PGE2 biosynthesis, has been associated with cancers of the colon [14,22,34], pancreas [16], stomach [17,35], post-menopausal breast [15,36], and lung [4]. However, this relationship was not observed in ovarian cancer [18], and our results now indicate very little evidence for elevated PGE-M playing a role in prostate cancer.

The finding of moderately decreased odds of prostate cancer in EA men with elevated PGE-M was unexpected given what is already known about elevated PGE-M being associated with cancer development at other organ sites such as the colon (summarized in Table S4). We do not have an answer as to why this may be happening, but what it does suggest is that COX signaling may be working differently in prostate cancer when compared to other cancer sites, with PGE2 signaling not being an oncogenic driver of the disease.

Chronic inflammation has been described as a prostate cancer risk factor that is associated with aggressive disease [2,3]. Epidemiological studies have reported protective effects of aspirin against aggressive disease and adverse outcomes in high-risk groups with prostate cancer [23,24]. Identifying PGE-M as a novel marker of aggressive disease would have importance for high-risk groups such as men of African descent who experience a disproportionately high burden of prostate cancer lethality. However, we did not find evidence for such a mechanism.

The lack of a robust PGE-M inhibition in both cases and controls who reported aspirin use in our study was surprising. Many studies have now demonstrated how aspirin use, at various doses, lowers urinary PGE-M levels [34,37]. However, Drew et al. reported approximately 20–25% of participants in the ASPIRED study who were randomized to 81 or 325 mg/day of aspirin experienced no inhibition or even an increase in PGE-M from baseline despite demonstrating a strong inhibition of urinary thromboxane B2 [22]. This finding is consistent with our data, where in a previous study, using the same cohort of participants, we reported strong thromboxane B2 inhibition in aspirin users across both cases and controls [38]. This suggests that the participants in our study are not non-responders to aspirin, but rather, aspirin use mechanistically is not targeting PGE-M in these men. It may also suggest that higher doses of aspirin are required in some men to achieve significant levels of PGE-M inhibition. Our lack of dose information prevents us from further investigating this phenomenon.

Our study has certain limitations. First, the case–control study design is retrospective, so it remains unknown if elevated PGE-M is protective against prostate cancer development in EA men. Second, we lack aspirin dose and dose compliance information, which prevents us from presenting data on the importance of aspirin dose in preventing all-cause mortality in AA men. However, the ASPIRED and AFPPS trials observed similar levels of PGE-M inhibition when either 81 mg/day or 325 mg/day doses were administered [22,37]. We also lack detailed data on the frequency of aspirin use, which would potentially provide more accurate estimates of PGE-M inhibition following aspirin use.

A major strength of this case–control study is the participant diversity, with very similar numbers of AA and EA participants. With 50% of cases and 47% of controls self-reporting as AA, we have a unique opportunity to examine biological factors that may promote cancer differently between population groups.

## 5. Conclusions

In summary, we evaluated associations between PGE-M levels and prostate cancer in a case–control study. We found no association between urinary PGE-M and aggressive prostate cancer. An association between elevated PGE-M and all-cause mortality in non-aspirin AA users reinforces the potential benefit of aspirin for the prevention of lethal prostate cancer, but the evidence does not support the mechanism of action to be via PGE-M inhibition. A prospective study is needed to confirm these findings.

## Figures and Tables

**Table 1:** Association of urinary PGE-M levels with prostate cancer.

Odds of Case Status *N*, (%)											
	All Cases			African American			European American				
PGE-M	Control	Case	OR (95% CI) *	Control	Case	OR (95% CI) *	Control	Case	OR (95% CI) *	P Heterogeneity	P Interaction
≤Median ^a^	499 (50)	482 (51)	Reference	231 (50)	216 (46)	Reference	268 (50)	266 (56)	Reference	0.03	0.04
>Median	499 (50)	466 (49)	0.86 (0.72–1.04)	234 (50)	256 (54)	1.0 (0.75–1.31)	265 (50)	210 (44)	**0.76 (0.59–0.99)**		
Continuous ^b^			0.96 (0.89–1.03)			1.03 (0.93–1.14)			**0.88 (0.79–0.98)**		

Note: column total sums (*N*, %) that differ are due to missing data. Bolded data indicate significant associations in the logistic regression analysis. ^a^ Median cutoff point is 14.22 ng PGE-M per mg creatinine. ^b^ PGE-M as a continuous, log2 transformed variable. * Unconditional logistic regression adjusted for age at study entry, BMI (kg/m^2^), diabetes (no/yes), aspirin (no/yes), education (high school or less, some college, college, professional school), family history of prostate cancer (first-degree relatives, yes/no), self-reported race (not included in stratified analysis), smoking history (never, former, current).

**Table 2:** Association of high urinary PGE-M with national comprehensive cancer network risk (NCCN) score for metastatic prostate cancer.

NCCN Risk Score	OR (95% CI) *	*p* Value
Low	Reference	
Intermediate	0.96 (0.65–1.43)	0.85
High/Very High	1.13 (0.71–1.81)	0.60
Regional/Metastatic	0.93 (0.44–1.97)	0.85

Note: * Unconditional logistic regression adjusted for age at study entry, BMI (kg/m^2^), diabetes (no/yes), aspirin (no/yes), education (high school or less, some college, college, professional school), family history of prostate cancer (first-degree relatives, yes/no), self-reported race, smoking history (never, former, current), treatment (0 = none, 1 = surgery, 2 = radiation, 3 = hormone, 4 = combination).

**Table 3:** Association of urinary PGE-M levels with all-cause mortality among prostate cancer patients.

Title	PGE-M	Alive	Dead	HR (95% CI)	HR (95% CI) ^†^	HR (95% CI) *
**All Cases**	≤Median ^a^	376 (53)	105 (44)	Reference	Reference	Reference
	>Median	329 (47)	135 (56)	**1.43 (1.11–1.84)**	**1.35 (1.05–1.74)**	1.16 (0.89–1.52)
	Continuous ^b^			**1.12 (1.02–1.24)**	1.09 (0.99–1.21)	1.00 (0.90–1.12)
**African American**	≤Median ^a^	165 (49)	50 (37)	Reference	Reference	Reference
	>Median	171 (51)	84 (63)	**1.47 (1.04–2.08)**	1.39 (0.98–1.96)	1.30 (0.91–1.87)
	Continuous ^b^			1.09 (0.96–1.23)	1.07 (0.94–1.23)	1.02 (0.88–1.17)
**European American**	≤Median ^a^	211 (57)	55 (52)	Reference	Reference	Reference
	>Median	158 (43)	51 (48)	1.26 (0.86–1.84)	1.14 (0.78–1.67)	0.97 (0.64–1.47)
	Continuous ^b^			1.12 (0.96–1.32)	1.05 (0.89–1.24)	0.95 (0.80–1.13)

Note: column total sums (*N*, %) that differ are due to missing data. Bolded data indicate significant associations in the Cox regression analysis. ^a^ Median cutoff point is 14.22 ng PGE-M per mg creatinine. ^b^ PGE-M as a continuous, log2 transformed variable. ^†^ Unconditional Cox regression adjusted for age at study entry. * Unconditional Cox regression adjusted for age at study entry, BMI (kg/m^2^), diabetes (no/yes), aspirin (no/yes), education (high school or less, some college, college, professional school), family history of prostate cancer (first-degree relatives, yes/no), self-reported race (not included in stratified analysis), smoking history (never, former, current), treatment (0 = none, 1 = surg, 2 = radiation, 3 = hormone, 4 = combination), disease stage (1 = stage I, 2 = stage IIA and IIB, 3 = stage III, and 4 = stage IV), Gleason score (0 = Gleason ≤ 7 and 1 = Gleason > 7).

**Table 4:** Association of urinary PGE-M levels with prostate cancer-specific mortality among prostate cancer patients.

Title	PGE-M	Alive	Death from PC	Death from Other Cause	HR (95% CI)	HR (95% CI) ^†^	Alive *	Death from PC *	Death from Other Cause *	HR (95% CI) *
**All Cases**	≤Median ^a^	388 (53)	28 (42)	80 (44)	Reference	Reference	375 (53)	27 (42)	78 (45)	Reference
	>Median	339 (47)	38 (58)	100 (56)	1.47 (0.90–2.41)	1.39 (0.85–2.28)	327 (47)	38 (58)	96 (55)	1.46 (0.86–2.46)
	Continuous ^b^				**1.24 (1.03–1.50)**	**1.23 (1.01–1.49)**				1.21 (0.97–1.50)
**African American**	≤Median ^a^	172 (49)	15 (38)	36 (38)	Reference	Reference	165 (49)	15 (38)	35 (37)	Reference
	>Median	178 (51)	25 (63)	60 (63)	1.43 (0.75–2.70)	1.33 (0.70–2.52)	171 (51)	25 (62)	59 (63)	1.49 (0.75–2.98)
	Continuous ^b^				1.18 (0.94–1.48)	1.17 (0.92–1.50)				1.13 (0.85–1.48)
**European American**	≤Median ^a^	216 (57)	13 (50)	44 (52)	Reference	Reference	210 (57)	12 (48)	43 (54)	Reference
	>Median	161 (43)	13 (50)	40 (48)	1.28 (0.58–2.81)	1.16 (0.53–2.56)	156 (43)	13 (52)	37 (46)	1.30 (0.54–3.14)
	Continuous ^b^				1.27 (0.91–1.78)	1.20 (0.85–1.70)				1.43 (0.95–2.15)

Note: column total sums (*N*, %) that differ are due to missing data. Bolded data indicate significant associations in the Cox regression analysis. ^a^ Median cutoff point is 14.22 ng PGE-M per mg creatinine. ^b^ PGE-M as a continuous, log2 transformed variable. ^†^ Unconditional Cox regression adjusted for age at study entry. * Unconditional Cox regression adjusted for age at study entry, BMI (kg/m^2^), diabetes (no/yes), aspirin (no/yes), education (high school or less, some college, college, professional school), family history of prostate cancer (first-degree relatives, yes/no), self-reported race (not included in stratified analysis), smoking history (never, former, current), treatment (0 = none, 1 = surg, 2 = radiation, 3 = hormone, 4 = combination), disease stage (1 = stage I, 2 = stage IIA and IIB, 3 = stage III, and 4 = stage IV), Gleason score (0 = Gleason ≤ 7 and 1 = Gleason > 7).

**Table 5:** Association of urinary PGE-M levels with all-cause mortality among men with prostate cancer after stratification by aspirin use.

Title	All Cases			African American				European American				
PGE-M	Alive	Dead	HR (95% CI) *	Alive	Dead	HR (95% CI) *	P Heterogeneity	Alive	Dead	HR (95% CI) *	P Interaction #	P Interaction ^†^
**Aspirin use**												
≤Median	182 (53)	62 (50)	Reference	61 (43)	25 (42)	Reference	0.04	121 (60)	37 (56)	Reference	0.11	0.38
>Median	162 (47)	63 (50)	0.92 (0.63–1.34)	81 (57)	34 (58)	0.70 (0.40–1.23)		81 (40)	29 (44)	1.05 (0.63–1.75)		
Continuous ^b^			0.93 (0.81–1.07)			0.85 (0.70–1.02)				1.00 (0.81–1.23)		
**No aspirin use**												
≤Median	193 (53)	43 (38)	Reference	104 (54)	25 (33)	Reference		89 (54)	18 (46)	Reference		
>Median	165 (46)	71 (62)	**1.52 (1.03–2.24)**	90 (46)	50 (67)	**2.04 (1.23–3.37)**		75 (46)	21 (54)	0.99 (0.48–2.07)		
Continuous ^b^			1.14 (0.96–1.35)			**1.27 (1.03–1.57)**				0.91 (0.67–1.22)		

Note: Bolded data indicate significant associations in the Cox regression analysis. ^a^ median cutoff point is 14.22 ng PGE-M per mg creatinine. ^b^ PGE-M as a continuous, log2 transformed variable. * Unconditional Cox regression adjusted for age at study entry, BMI (kg/m^2^), diabetes (no/yes), education (high school or less, some college, college, professional school), family history of prostate cancer (first-degree relatives, yes/no), self-reported race (not included in stratified analysis), smoking history (never, former, current), treatment (0 = none, 1 = surgery, 2 = radiation, 3 = hormone, 4 = combination), disease stage (1 = stage I, 2 = stage IIA and IIB, 3 = stage III, 4 = stage IV), Gleason score (0 = Gleason ≤ 7 and 1 = Gleason > 7) and stratified by aspirin (no/yes). # Test for interaction between level of PGE-M and aspirin use. ^†^ Test for interaction between self-reported race and aspirin use.

## Data Availability

The data underlying this article will be shared upon reasonable request to the corresponding author.

## Supplementary Materials

The following are available online at https://www.mdpi.com/article/10.3390/cancers13164073/s1, Supplementary Methods, Table S1: Characteristics of cases and population controls in the NCI–Maryland Prostate Cancer Case–Control Study, Table S2: Men with prostate cancer by urinary PGE-M level and national comprehensive cancer network risk score, Table S3: Association of regular aspirin use with urinary PGEM levels in cases and controls, Table S4: Association of PGE-M and cancer risk from published literature, Figure S1: High urinary PGE-M levels are not significantly associated with increased prostate cancer-specific mortality.
