# Supplementary material for: Urinary PGE-M in Men with Prostate Cancer
Source: Cancers (Basel). 2021 Aug 13;13(16):4073. doi: 10.3390/cancers13164073 (PMC8391815; doi:10.3390/cancers13164073)
Supplement: Supplementary file 1 [file cancers-13-04073-s001.zip › supplementary.pdf]

Supplemental Methods

Study Population

Eligibility criteria for the NCI-Maryland prostate cancer case-control study included the following: diagnosis with prostate cancer within two years prior to enrollment; residence in Maryland or adjacent counties in Pennsylvania, Delaware, Virginia, or District of Columbia; 40 to 90 years old at the time of enrollment and born in the United States; either African American or European American by self-report; can be interviewed in English; had a working home phone number; be physically and mentally fit to be interviewed; not severely ill and not residing in an institution such as prison, nursing home, or shelter. Cases were recruited within 2 years after disease diagnosis with median interval between diagnosis and enrollment of 4.9 months. The controls had the same eligibility criteria as cases with the exception that they could not have a personal history of cancers other than non-melanoma skin cancer, radiation therapy, or chemotherapy.

At the time of enrollment, both cases and controls were administered a questionnaire by a trained interviewer. The interviewer asked about their demographics, tobacco use, nutrition, medical history, family history of cancer, prostatitis, or benign prostatic hypertrophy, occupational history, socioeconomic status, anthropometry, and sexual history. Blood, urine, and mouthwash rinse/buccal cells samples were collected from each participant.

Laboratory assay for urinary PGE-M measurement.

PGE-M was measured by the Eicosanoid Core Laboratory at Vanderbilt University Medical Center (Nashville, TN) (principal investigator, Ginger Milne) using a LC/MS method that has been previously described [1, 2].

The sample was dried under a stream of dry nitrogen at 37°C and then reconstituted in 75µl mobile phase A for liquid chromatography–mass spectrometry (LC-MS) analysis. LC was performed on a 2.0 x 50 mm, 1.7µm particle Acquity BEH C18 column (Waters Corporation, Milford, MA, USA). Mobile phase A was 95:4.9:0.1 (v/v/v) water: acetonitrile: acetic acid, and mobile phase B was 10.0:89.9:0.1 (v/v/v) water: acetonitrile: acetic acid. Samples were separated by a gradient of 85–5% of mobile phase A over 14 min at a flow rate of 0.375 mL/min prior to delivery to a SCIEX 6500+ QTrap mass spectrometer.

All urine samples (n=1999) were measured for PGE-M. These samples had previously been stored at −80°C. The limit of detection for PGE-M with the assay is about 0.025 ng/mL. At this cutoff level, 4/977 cases and 3/1022 controls were reported to have PGE-M levels below the lower limit of detection (LLOD). Urinary creatinine was used for standardization and measured using a test kit from Enzo Life Sciences, Inc. Levels of PGE-M were standardized to ng PGE-M per mg creatinine. All LLOD samples were kept in the analysis and set at the threshold of 0.025 ng PGE-M per mg creatinine for the analysis, which is lower than the lowest measured sample (0.028 ng PGE-M per mg creatinine). Urine from two cases and two controls was not detected so could not be included in the analysis. Median PGE-M levels with their interquartile range showed the following distribution in the study population: all cases: 14.03 (15.12); AA cases: 15.32 (15.74); EA cases: 12.56 (14.22); all controls: 14.22 (14.81); AA controls: 14.38 (14.67); EA controls: 14.2 (14.77).

With measurements completed over a period of months, cases and controls were randomized across batches and 5% quality control (QC) samples were included to assess analytic error for duplicates across plates ("inter-plate duplicates"). In this study, % CV for inter-plate duplicates was 9.6%. Laboratory personnel were blinded to case, control, and QC status for both the pilot and full study.

Multivariable logistic and Cox regression analyses - covariates

In the main analysis, investigating the association of urinary PGE-M levels with prostate cancer and survival, we adjusted the unconditional logistic regression and Cox proportional hazards models for the following potential confounding factors: age at study entry, body-mass index (BMI, kg/m^2), diabetes (no/yes), aspirin use (no/yes), education (high school or less, some college, college, professional school), family history of prostate cancer (first-degree relatives, yes/no), self-reported

race, smoking history (never, former, current), treatment (0=none, 1=surgery, 2=radiotherapy, 3=hormone, 4=combination), disease stage (1=stage I, 2=stage IIA and IIB, 3=stage III, & 4=stage IV), and Gleason score (0=Gleason ≤ 7 and 1=Gleason > 7).、

Supplementary Table S1. Characteristics of cases and population controls in the NCI-Maryland Prostate Cancer Case-Control Study.

Title	Cases[a]			Population Controls		
Demographics	All (n=977)	AA[b] (n=490)	EA[c] (n=487)	All (n=1,022)	AA (n=479)	EA (n=543)
**Age[d]**						
Median (IQR[e]) in years	64 (11)	63 (10)	65 (11)	65 (12)	64 (10)	66 (13)
**BMI**						
Median (IQR[e]) in kg/m^2	27.6 (5.7)	27.8 (6.8)	27.5 (5.2)	28.2 (6.9)	29.0 (7.2)	27.4 (6.5)
**Education, N (%)**						
High school or less	357 (37)	232 (47)	125 (26)	241 (24)	137 (29)	104 (19)
Some college	296 (30)	168 (34)	128 (26)	260 (25)	140 (29)	120 (22)
College	173 (18)	58 (12)	115 (24)	257 (25)	103 (21)	154 (28)
Graduate	141 (14)	27 (6)	114 (23)	251 (25)	90 (19)	161 (30)
Did not provide	10 (1)	5 (1)	5 (1)	13 (1)	9 (2)	4 (1)
**Family history of prostate cancer[f], N (%)**						
No	861 (88)	434 (89)	427 (88)	942 (92)	442 (92)	500 (92)
Yes	102 (11)	48 (10)	54 (11)	68 (7)	29 (6)	39 (7)
Did not provide	14 (1)	8 (1)	6 (1)	12 (1)	8 (2)	4 (1)
**Smoking status[g], N (%)**						
Current	242 (25)	166 (34)	76 (16)	148 (15)	95 (20)	53 (10)
Former	396 (41)	177 (36)	219 (45)	462 (45)	201 (42)	261 (48)
Never	324 (33)	139 (28)	185 (38)	402 (39)	180 (37)	222 (40)
Did not provide	15 (1)	8 (2)	7 (1)	10 (1)	3 (1)	7 (2)
**Diabetes, N (%)**						
No	748 (77)	344 (70)	404 (83)	768 (75)	326 (68)	442 (81)
Yes	218 (22)	141 (29)	77 (16)	242 (24)	145 (30)	97 (18)
Did not provide	11 (1)	5 (1)	6 (1)	12 (1)	8 (2)	4 (1)
**Disease Characteristics**						
**Stage[h], N (%)**						
T1	188 (19)	77 (15)	111 (22)			
T2	646 (66)	351 (70)	295 (61)			
T3	82 (8)	27 (5)	55 (11)			
T4	59 (6)	34 (7)	25 (5)			
Did not provide	2 (<1)	1 (3)	1 (<1)			
**Gleason score, N (%)**						
≤7	406 (42)	192 (39)	214 (44)			
>7	571 (58)	298 (61)	273 (56)			
**PSA[i]**						
Median (IQR[e]) in ng/ml	6.34 (5.9)	7 (7.76)	6 (4.6)			
**Treatment, N (%)**						
None	83 (8)	48 (10)	35 (7)			
Surgery	185 (19)	90 (18)	95 (20)			
Radiotherapy	291 (30)	154 (32)	137 (28)			

Hormone	50 (5)	39 (8)	11 (2)
Combination	252 (26)	129 (26)	123 (25)
Did not provide	116 (12)	30 (6)	86 (18)

Note: a Cases recruited within 2 years after disease diagnosis with an average interval between diagnosis and enrollment of 6.7 months b AA: African American c EA: European American d Age at study interview e IQR: Interquartile range f First-degree relative with prostate cancer g Smoking status describes cigarette smoking h Pathologically confirmed using American Joint Committee on Cancer (AJCC) 7th Edition i PSA: Prostate specific antigen.

Supplementary Table S2. Men with prostate cancer by urinary PGE-M level and National Comprehensive Cancer Network Risk Score.

Title	PGE-M ≤ Median N (%)	PGE-M > Median N (%)	Total
Low	97 (52)	90 (48)	187 (100)
Intermediate	255 (51)	250 (50)	505 (100)
High	116 (51)	112 (49)	228 (100)
Regional/Metastatic	27 (52)	25 (48)	52 (100)
Total	495 (51)	477 (49)	972 (100)

Supplementary Table S3. Association of regular aspirin use with urinary PGEM levels in cases and controls.

Title	Aspirin Use N, (%)								
Title	All Cases			African American			European American		
PGEM	No	Yes	OR (95% CI) *	No	Yes	OR (95% CI) *	No	Yes	OR (95% CI) *
Q1[a]	117 (25)	130 (28)	Reference	64 (24)	40 (20)	Reference	53 (26)	90 (33)	Reference
Q2	120 (25)	114 (24)	0.79 (0.52-1.13)	65 (24)	46 (23)	0.98 (0.45-1.75)	55 (27)	68 (25)	0.69 (0.40-1.19)
Q3	123 (26)	109 (24)	0.66 (**0.45-0.89**)	72 (27)	55 (27)	0.99 (0.56-1.76)	51 (25)	54 (20)	0.48 (**0.27-0.84**)
Q4	115 (24)	118 (25)	0.75 (0.50-1.12)	69 (26)	60 (30)	1.09 (0.61-1.93)	46 (22)	58 (21)	0.53 (**0.29-0.94**)
			P trend 0.11			P trend 0.73			**P trend 0.01**
Continuous[b]			0.94 (0.85-1.05)			1.07 (0.92-1.24)			**0.81 (0.69-0.96)**
	All Controls			African American			European American		
PGEM	No	Yes	OR (95% CI) †	No	Yes	OR (95% CI) †	No	Yes	OR (95% CI) †
Q1	96 (22)	153 (27)	Reference	52 (23)	63 (27)	Reference	44 (22)	90 (27)	Reference
Q2	109 (25)	141 (25)	0.82 (0.57-1.20)	57 (25)	59 (25)	0.88 (0.51-1.54)	52 (26)	82 (25)	0.68 (0.40-1.16)
Q3	118 (27)	135 (24)	0.73 (0.50-1.07)	62 (27)	55 (23)	0.75 (0.43-1.31)	56 (28)	80 (24)	0.63 (0.37-1.07)
Q4	109 (25)	137 (24)	**0.60 (0.40-0.89)**	58 (25)	59 (25)	0.60 (0.34-1.07)	51 (25)	78 (24)	**0.55 (0.31-0.96)**
			**P trend 0.01**			P trend 0.07			**P trend 0.03**
Continuous[b]			**0.85 (0.86-0.96)**			0.88 (0.76-1.03)			**0.79 (0.65-0.95)**

Note: Bolded data indicate significant associations in the logistic regression analysis. a Quartile (Q) cutoff points are 8.12, 14.24 and 22.98 ng PGEM per mg creatinine.b PGEM as a continuous, log2 transformed variable. *Logistic regression analysis adjusted for age at study entry, BMI (kg/m2), diabetes (yes/no), education (high school or less, some college, college, professional school), family history of prostate cancer (first-degree relatives, yes/no), self-reported race (not included in stratified analysis), disease stage (1 = stage I, 2 = stage IIA and IIB, 3 = stage III, & 4 = stage IV), Gleason score (0 = Gleason ≤ 7 and 1 = Gleason >7), smoking history (never, former, current), treatment (0=none, 1=surgery, 2=radiation, 3=hormone, 4=combination) † Logistic regression analysis adjusted for age at study entry, BMI (kg/m2), diabetes (yes/no), education (high school or less, some college, college, professional school), family history of prostate cancer (first-degree relatives, yes/no), self-reported race (not included in stratified analysis), smoking history (never, former, current).

Supplementary Table S4. Association of PGE-M and cancer risk from published literature.

Cancer Site	Positive association with cancer risk	Reference
Breast cancer	Yes	[3]
Colorectal cancer	Yes	[4]
Gastric cancer	Yes	[5]
Lung cancer	Yes	[6]
Ovarian cancer	No	[2]
Pancreatic cancer	Yes	[7]

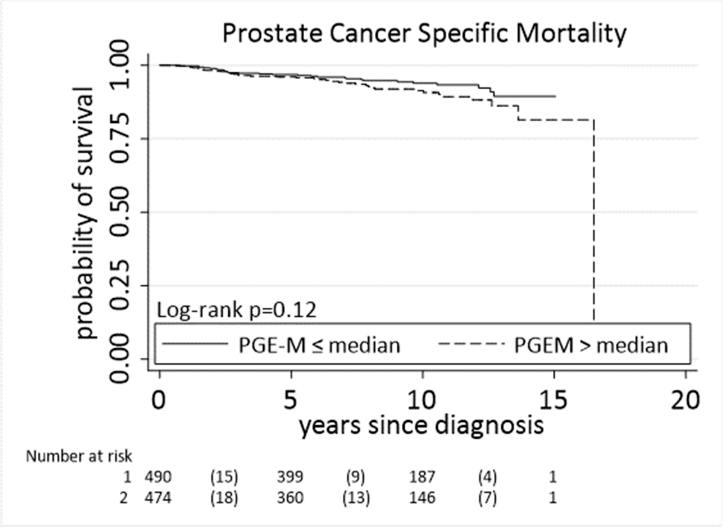

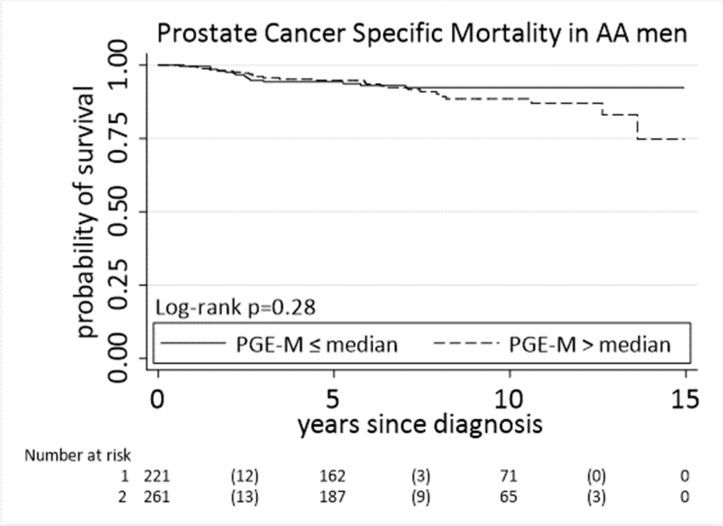

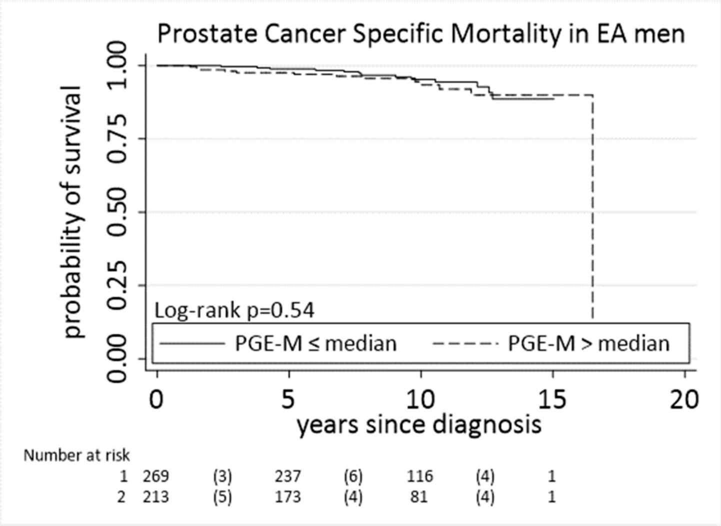

Supplementary Figure S1. High urinary PGE-M levels are not significantly associated with increased prostate cancer-specific mortality. Kaplan–Meier survival plot showing association of dichotomized PGE-M levels with prostate cancer-specific mortality among all cases (A) and after stratification into African American (AA) (B) and in European American (EA) men (C). High PGE-M levels: ≥ median.

Supplementary References

1. Duffield-Lillico AJ, Boyle JO, Zhou XK, *et al.* Levels of prostaglandin E metabolite and leukotriene E4 are increased in the urine of smokers: evidence that celecoxib shunts arachidonic acid into the 5-lipoxygenase pathway. Cancer Prevention Research 2009;2(4):322-329.
2. Barnard ME, Beeghly-Fadiel A, Milne GL, *et al.* Urinary PGE-M levels and risk of ovarian cancer. Cancer Epidemiology and Prevention Biomarkers 2019;28(11):1845-1852.
3. Kim S, Campbell J, Yoo W, *et al.* Systemic levels of estrogens and PGE2 synthesis in relation to postmenopausal breast cancer risk. Cancer Epidemiology and Prevention Biomarkers 2017;26(3):383-388.
4. Johnson JC, Schmidt CR, Shrubsole MJ, *et al.* Urine PGE-M: a metabolite of prostaglandin E2 as a potential biomarker of advanced colorectal neoplasia. Clinical Gastroenterology and Hepatology 2006;4(11):1358-1365.
5. Wang T, Cai H, Zheng W, *et al.* A prospective study of urinary prostaglandin E2 metabolite, helicobacter pylori antibodies, and gastric cancer risk. Clinical Infectious Diseases 2017;64(10):1380-1386.
6. Murphey LJ, Williams MK, Sanchez SC, *et al.* Quantification of the major urinary metabolite of PGE2 by a liquid chromatographic/mass spectrometric assay: determination of cyclooxygenase-specific PGE2 synthesis in healthy humans and those with lung cancer. Analytical biochemistry 2004;334(2):266-275.
7. Zhao J, Wang J, Du J, *et al.* Urinary Prostaglandin E 2 Metabolite and Pancreatic Cancer Risk: Case-Control Study in Urban Shanghai. PloS one 2015;10(2):e0118004.
